# Healthcare Professionals’ Perspectives on the Cross-Sectoral Treatment Pathway for Women with Gestational Diabetes during and after Pregnancy—A Qualitative Study

**DOI:** 10.3390/jcm10040843

**Published:** 2021-02-18

**Authors:** Anne Timm, Karoline Kragelund Nielsen, Ulla Christensen, Helle Terkildsen Maindal

**Affiliations:** 1 Health Promotion Research, Steno Diabetes Center Copenhagen, 2820 Gentofte, Denmark; karoline.kragelund.nielsen@regionh.dk (K.K.N.); htm@ph.au.dk (H.T.M.); 2 Department of Public Health, University of Copenhagen, 1014 Copenhagen, Denmark; ulch@sund.ku.dk; 3 Department of Public Health, Aarhus University, 8000 Aarhus, Denmark

**Keywords:** qualitative research, gestational diabetes mellitus, type 2 diabetes mellitus, maternal health, healthcare system, lifestyle, risk reduction, diabetes prevention, obesity prevention

## Abstract

Gestational diabetes mellitus (GDM) increases the risk of adverse outcomes during and after pregnancy, including a long-term risk of type 2 diabetes. Women with GDM are treated by numerous healthcare professionals during pregnancy and describe a lack of preventive care after pregnancy. We aim to investigate healthcare professionals’ perspectives on the cross-sectoral treatment pathway for women with GDM—during and after pregnancy. A qualitative study was conducted using systematic text condensation. Nine healthcare professionals (two general practitioners, four midwives, two obstetricians and one diabetes nurse) were interviewed and eight health visitors participated in two focus group discussions., Three major themes emerged: (1) “professional identities”, which were identified across healthcare professionals and shaped care practices; (2) ”unclear guidelines on type 2 diabetes prevention after GDM”, which contributed to uncertainty about tasks and responsibilities during and after pregnancy; and (3) “cross-sectoral collaboration”, which relied heavily on knowledge transfers between hospitals, general practice and the local municipality. The findings implicate that clear, transparent guidelines for all sectors should be prioritized to strengthen cross-sectoral care to women with GDM during and after pregnancy. As a result, strong cross-sectoral care throughout the GDM care pathway may improve maternal health by supporting healthy behaviors, facilitate weight loss and reduce the risk of subsequent GDM and early onset diabetes.

## 1. Introduction

Gestational diabetes mellitus (GDM) is a transient condition affecting 1.8–31.5% of pregnant women worldwide [1]. In Denmark, 2–4% of deliveries are affected by GDM [2,3] The main risk factors for developing GDM include maternal age, family history of diabetes, ethnicity, excess weight gain in pregnancy and maternal BMI [4,5] Among women giving birth in Denmark in the period from 2004 to 2015, 70% of the women who developed GDM were overweight or obese pre-pregnancy [6] GDM is associated with an increased risk of several complications affecting both mother and offspring during pregnancy and delivery [7].Due to the “acuteness” of the condition, women with GDM are closely followed by multiple healthcare professionals (HCPs) in pregnancy. In Denmark, this includes midwives, obstetricians, endocrinologists, general practitioners (GPs) and dieticians (See Table 1). After delivery, most women with prior GDM return to a normoglycemic stage but remain at high risk of developing type 2 diabetes [8]. In addition, women with prior GDM often develop GDM in subsequent pregnancies [2]. Identifying strategies for prevention of type 2 diabetes and recurrent GDM has therefore been a key research priority in recent decades. BMI is the main modifiable risk factor for type 2 diabetes after GDM [9], and structured lifestyle interventions for women with prior GDM have proved to be effective in preventing or postponing diabetes onset through weight loss [10]. Regular glucose testing and follow-up focusing on achieving a healthy lifestyle postpartum are therefore recommended in order to prevent or delay and detect diabetes development in a timely manner. After delivery, the “care” paradigm shifts to a more long-term, non-acute prevention focus. According to Danish national guidelines, the GP has the primary responsibility for regular glucose testing and counseling on healthy lifestyle following a GDM-affected pregnancy [11,12]. However, studies indicate that this follow-up does not occur extensively; that care is not always tailored to individual needs and preferences; and that coordination of services and responsibility is unclear or lacking [13,14,15]. Additionally, women with prior GDM find information on risks and recommendations from the GP inadequate and often do not follow lifestyle recommendations after delivery [16,17]. We have previously shown that Danish women with prior GDM experience treatment in pregnancy as strict and uncomfortable and are further challenged by the baby’s needs, lack of support from both HCPs and their partner to engage in health behaviors postpartum [17].

In this study, we investigate HCPs’ perspectives on the cross-sectoral treatment pathway for women with GDM during and after pregnancy.

## 2. Materials and Methods

### 2.1. Study Design and Data Collection

This is a qualitative study using focus group discussions and individual semi-structured interviews. Focus group discussions were conducted exploratively to gain background knowledge on experiences with treatment of GDM and to stimulate new research hypotheses [18]. Themes identified in the focus group discussions acted as context and support for the semi-structured interviews that were subsequently carried out with other HCPs.

We held two focus group discussions with four health visitors each from the Greater Copenhagen (Capital) area to explore their knowledge and experience with women with prior GDM. The focus group discussions relied on open-ended questions, as this allows for multiple responses and inspires different interpretations [19].The topics in the interview guides concerned health visitors’ experiences with women and families with prior GDM, including questions such as: “What do you think is important to discuss with families where the mother had gestational diabetes?”, “In your opinion, what can be done to optimize care for this group with the current resources?” and “How should postpartum care for women with prior gestational diabetes be?”.

The questions in the semi-structured interviews were inspired by Spradley wherefore descriptive and structural questions were assessed before every new interview as the profession and setting changed [20]. The interview guide focused on organizational settings, cross-sectoral collaboration and possibilities for intervention in the postpartum period (See Table S1). For example, questions relating to the HCP’s interest and motivation for interprofessional coordination were addressed: “Which healthcare professionals are you planning the follow-ups after pregnancy with?” and “How would you like the collaboration to be?”. The approach was explorative, and the interview guide was adapted throughout the study to allow exploration of new themes arising.

### 2.2. Participants and Study Settings

A purposive sampling strategy was used to recruit various HCPs providing care to women with GDM during and after pregnancy to represent the full GDM care trajectory (Table 1). Diabetes nurses, midwives, endocrinologists, obstetricians, dieticians and GPs were invited by email and/or phone call, introducing the study aim and proposing interview dates. Moreover, health visitors who provide health-promoting care to all Danish families during the postpartum period were invited to participate in focus group discussions. In addition to contacting potential informants by email and/or phone call, we used a snowball method asking participating HCPs to contact colleagues. When no new perspectives came up in interviews, data saturation was reached, and we stopped recruiting new participants.

### 2.3. Analysis

Focus group discussions and interviews were audio recorded and transcribed verbatim. We analyzed the text using systematic text condensation (STC) [21], which ensures a structured, transparent analysis. The method is rooted in phenomenological analysis and is inspired by iterative processes of “decontextualization” and “recontextualization”. The purpose of STC is to understand informants’ experiences and perspectives and seeks to be data-driven in its approach. STC further advances analysis by enabling identification of core themes and essential characteristics of data [21]. The semi-structured interviews were analyzed in five steps; (1) chaos to themes, (2) sorting meanings, (3) condensation, (4) synthesizing and (5) sequencing [21]. Transcripts from the interviews were first coded into categories and then into themes in an iterative process. The five steps of STC were repeated until the major themes covered the HCPs’ perspectives. Nvivo 11 (QSR International Pty Ltd., Loncaster, Australia) was used to assist the coding and analysis of the transcripts.

### 2.4. Ethical Considerations

As the study relies on qualitative methods, it is exempt from ethical approval according to the regulations of The Danish National Committee on Health Research Ethics. Oral consent was collected from participants in the interviews and focus group discussions [22]. Throughout the interviews, unintentional consequences of the interviews and focus group discussions were considered [23].

## 3. Results

Two focus group discussions were carried out with a total of eight health visitors from a municipality in the Copenhagen area in Denmark. Further, semi-structured interviews were conducted with a total of nine HCPs—four midwives, two obstetricians, two GPs and one diabetes nurse from obstetric departments at hospitals in Aarhus and Copenhagen in Denmark. The study participants represent professions from general practice, obstetric departments and municipal healthcare services. The characteristics of the participants are presented in Table 2.

Three major themes emerged from the analysis: (1) professional identities, (2) unclear guidelines on type 2 diabetes prevention after GDM and (3) cross-sectoral collaboration (Table 3).

### 3.1. Professional Identities

The HCPs had different tasks and assignments in their interaction with women with GDM. Obstetricians were first and foremost responsible for treating short-term consequences associated with a GDM-affected pregnancy, and thereafter responsible for counseling women with GDM about the excess risk of type 2 diabetes after pregnancy. It was most important to the obstetricians to communicate the potential “acute” consequences during pregnancy.

“We talk about the risk of birth complications; the importance of keeping normal blood sugars because that is what they especially need to take care of. I also tell them that there is already a risk related to their children becoming overweight and developing metabolic disturbances in childhood and emphasize the importance of thinking about diet and exercise for their children”.

(Obstetrician, N)

The diabetes nurse also viewed her profession as critical in disseminating knowledge about the implications of GDM on the body and more practically how to measure blood sugar levels.

“They [women diagnosed with GDM] receive a basic knowledge about what happens in the body to make them well-informed. Also, that it is important to keep an eye on their blood sugars in pregnancy and we teach them how to do blood sugar measurements”.

(Diabetes Nurse, P)

The obstetricians and the diabetes nurse underlined the importance of reinforcing dietary restrictions during pregnancy to ensure that the health of the baby was prioritized. They indicated that different professional identities across the GDM care trajectory created natural opportunities for midwives to take care of the women and the emotional burden they might feel about the GDM regime.

“The midwives try to hold on to the pregnancy course and baby and delivery and family formation, so they try to stay away from the sick because that’s what we take care of. We deal with it [GDM] both as dietitians and as nurses. And many of the patients actually report that it is such a nice ‘refuge’ [in quote] to consult with a midwife”.

(Diabetes Nurse, P)

The midwives similarly reported focusing on the health of the fetus, but also attending to the health of the mother in a more general sense. The conversation between the mother and the midwife comprised the woman presenting her narrative about her pregnancy. A midwife pointed out how it helped to motivate the women to make healthy decisions during pregnancy.

“I always make them reflect on what makes them overweight. Also, I help and motivate them in terms of where there is something they can work with”.

(Midwife, I)

The midwives’ described feeling inclined to support the women to cope with the pregnancy affected by GDM. One midwife even used the term “pregnancy prison” to illustrate the strict regimen women with GDM had to follow during pregnancy.

“So, they [women with GDM] feel that they are in a rather pregnancy prison-like state. And when they get rid of all the controls they have during pregnancy, [they feel like] “they can live their lives completely free again”. Then they really forget what motivated them to hold on to the good habits”.

(Midwife, K)

Health visitors noted that their key role was to ensure the wellbeing of the baby and facilitate a healthy family dynamic. They perceived themselves as having a close relationship with women after a GDM-affected pregnancy as they interact with the woman outside the hospital setting. Still, the health visitors expressed having limited knowledge about GDM and described that they comforted the women by telling them that developing type 2 diabetes was out of the women’s control.

“We try to hold on to the fact that it is because of genes for the most part. And they can’t do anything about that. Not that we want to take the responsibility away from them, but it does no good that you walk around feeling guilty”.

(Health visitor, A)

Finally, GPs are in contact with women with GDM both during pregnancy and after delivery. The GPs stated that during pregnancy, it was up to the women to decide what to discuss during the consultations, as they did not want to burden the women further by focusing on the long-term risks associated with GDM. When one of the GPs was asked if he would initiate a conversation about the implications of a GDM diagnosis for mother and child, he replied:

“No, I do not think so. […] It is the pregnant women who sets the agenda. So, sitting and giving long speeches—we don’t do that. If she is worried then we will talk about it, but there is a lot to be done in the consultation that does not necessarily relate to gestational diabetes”.

(GP, Q)

Thus, the GPs, midwives and health visitors described difficulties addressing health behaviors as diet and physical activity relevant to a healthy lifestyle following a GDM diagnosis. However, the obstetricians and diabetes nurse mainly talked about risk with the women as they perceived risk to be their main focus. The midwives did not want to blame the women by addressing the GDM-related health risks for the child as they were already provided with overwhelming amounts of information in pregnancy. Often, health visitors did not even know about the implications of a GDM-related pregnancy, while there was no reason for them to start conversations about diabetes risk or prioritize a focus on health behaviors after delivery. GPs focused on addressing other relevant topics, making women’s risk unlikely to be included in the consultation. Thus, health behaviors (and long-term prevention) were rarely discussed, as their discussion depended on whether the women brought them up herself, and whether it was deemed appropriate by the individual HCP to prioritize the topic in the consultation.

### 3.2. Unclear Guidelines on Type 2 Diabetes Prevention after GDM

A concern that was widely pronounced by HCPs in the obstetric departments was the lack of importance given to the prevention of type 2 diabetes after a GDM-affected pregnancy resulting in lack of or unclear guidelines in the transition from pregnancy to the postpartum period. The HCPs also reported that the lack of resources allocated to preventive care after GDM reduced their flexibility in working with women with GDM in pregnancy. Additionally, it created a feeling that the management was not supportive of HCPs working with long-term implications of GDM.

“I’m not sure it’s that prestigious to work with it [GDM]. It is when they are pregnant, but afterwards I don’t think it’s that prestigious. I just think we don’t have enough focus on it. I don’t think it’s prioritized enough”.

(Obstetrician, O)

According to the HCPs, the limited follow-up of women with prior GDM reduced the possibility of upholding a supportive treatment system. For example, the diabetes nurse expressed that the focus on treatment during pregnancy neglected the need for long-term health promotion after delivery.

“Somehow, we cannot really in decency let them [women with GDM] go. I mean, it is a lot about the ‘treating’ healthcare system in a way. Where we are saying ‘okay now we have treated you and now there is no more, now you have to take care of yourself’. We need some more health promotion and prevention, also in the postpartum period”.

(Diabetes Nurse, S)

The GPs reported that after delivery, the primary focus of care shifts from the woman with GDM to the baby. They believed this shift to be partly due to a lack of guidelines on how to communicate the long-term risks associated with prior GDM to women after delivery.

“These women with gestational diabetes, they disappear a little alongside so many other things. There is a lot of focus during pregnancy, but after pregnancy, it disappears into the wellbeing of the baby and illness and so on”.

(GP, R)

“I think a precise to-do list for gestational diabetes is missing. We should focus on how we most appropriately can follow these women.‘ This was a case of gestational diabetes, but what then?’ It becomes forgotten”.

(GP, Q)

The HCPs described that the guidelines for prevention of type 2 diabetes after GDM were ill-prioritized when women “lost” their GDM diagnosis. The HCPs who attended women in the obstetric departments (obstetrician, diabetes nurse and midwives) expressed a frustration with the lack of additional care in the period after delivery. They saw the lack of follow-up care as conflicting with their roles as caregivers, when they did not feel that their care for women with GDM in pregnancy was supported throughout the postpartum period. The GPs confirmed that the diagnosis of GDM was forgotten after pregnancy and called for clear guidelines on how to address the implications of GDM after delivery.

### 3.3. Cross-Sectoral Collaboration

HCPs’ collaboration was to a large extent described as being unstructured due to unclear responsibilities in information transfer between providers, particularly when information about women with GDM needed to travel across sectors, e.g., from the obstetric departments to GPs or health visitors. Barriers for cross-sectoral collaboration included: working in other hospital departments with no natural day-to-day interaction; and not finding information from the other HCPs relevant to their own practice. The midwives specified that interactions with other HCPs benefited collaboration by reducing repetition and losing important knowledge in the consultation. Nonetheless, only a few of the midwives collaborated closely with the outpatient clinic, and none of the HCPs worked in physical proximity to the GPs.

“Since we’re placed in two geographically different locations, it is hard to have a close collaboration. Of course, we read each other’s notes, but I know that the pregnant women experience that we, as healthcare professionals, say different things. Since I visit the hospital regularly, I don’t think that I communicate that differently from the professionals over there [obstetricians, dieticians, endocrinologists]. […] Because I go there [to the outpatient clinic] and have the possibility to talk to them [HCPs at the outpatient clinic]”.

(Midwife, K)

The midwives perceived the lack of information flow between HCPs as a serious challenge as much time was spent catching up on the woman’s special needs in the consultation. Thus, important information on the woman’s medical and social circumstances was lost in the knowledge transfer between providers.

“Yes, I think you distance yourself from what you don’t know that much about. Then you think: ‘they [the outpatient clinic] take care of that over there,’ and I take care of mine according to what I usually do”.

(Midwife, J)

According to the HCPs, divergent messages from dieticians and obstetricians on health risk caused women with GDM to neglect their elevated risk of type 2 diabetes after pregnancy. The HCPs explained how the quality of the information transfer between HCPs largely depended on individual reporting practices. For example, health visitors reported lacking information on whether the woman had GDM in her latest pregnancy as obstetric departments sometimes did not include it in the correspondence letter. As such, interacting with other HCPs encouraged coherent communication and awareness of other HCPs’ assignments. The diabetes nurse, midwives and health visitors explicitly stated that they were unsatisfied with their communication with the GPs.

“My own doctor just said I have to avoid putting sugar in the coffee’ [referring to a statement from a woman with GDM]. There is a big difference between what they are told by their GP and what we do. We find that there are many practitioners who neglect that young fertile women may develop type 2 diabetes”.

(Diabetes Nurse, P)

“You have to keep the doctors in general practices on their toes. So, they [women with prior GDM] know what they have to go through. Then, the women would tell their doctors: ‘Excuse me, but I haven’t received information about a glucose tolerance test’, or something like that”.

(Health visitor, C)

The lack of information would sometimes become clear to GPs and health visitors in the consultations and during home visits after pregnancy, respectively, where the women often had to be the ones to tell the GP and health visitor about their GDM diagnosis. However, as with health visitors, GPs noted that they were not always provided with adequate information from the obstetric department about the latest pregnancy to better support and guide the women after a GDM diagnosis. GPs’ understanding of how GDM affects new mothers emotionally was also reported to be poor compared to other types of HCPs, suggesting a need for training of GPs and a strengthening of communication across sectors.

“I think what I need is a clearer handover of what the task is. What has been said in the diabetes or obstetric departments to these women. And then an early indication of how they should look after themselves and what is the appropriate way to follow up on that”.

(GP, Q)

It was essential for HCPs in the obstetric department to inform women about their future risk of type 2 diabetes. However, different communication forms across professions could cause women to perceive their GDM diagnosis as either demanding a lot of changes in everyday life or amenable with just a few changes. For women with GDM, who did not perceive the diagnosis to be of great importance, the diabetes nurse and obstetricians felt a need to change the women’s perspective to ensure that the diagnosis was taken seriously. Various communication strategies across sectors and limited information flow between HCPs resulted in poor cross-sectoral collaboration and follow-up. GPs reported that they were not provided with adequate information from the obstetric departments on the women’s treatment course, while health visitors experienced that women were uncertain about their future contact with the healthcare system. With missing information on prior GDM status, health visitors who visit women with prior GDM after delivery lose the opportunity to include GPs in restructuring and aligning cross-sectoral communication practices.

## 4. Discussion

Our study identified three themes describing HCPs’ perspectives on the cross-sectoral GDM care pathway: (1) professional identities, (2) unclear guidelines on type 2 diabetes prevention after GDM and (3) cross-sectoral collaboration (Table 3).

Women with GDM interact with several types of HCPs, each with specific professional identities, which demands strong collaboration between HCPs to secure a coherent treatment pathway. In our study, we found that when HCPs consulted women with GDM, HCPs often did not address risk and long-term health and lifestyle for various reasons. Obstetricians and the diabetes nurse prioritized the short-term implications of GDM during pregnancy and relied on other HCPs to engage in type 2 diabetes prevention after delivery. Midwives and health visitors were concerned about potentially compromising the relationship to the women with GDM by addressing risks associated with an unhealthy lifestyle. The GPs had other agendas in the consultations than GDM-related risk and healthy lifestyle, relying on the woman to take up the subject of diabetes prevention herself. HCPs working in obstetric departments reported that GDM-relevant long-term preventive care was neglected in the healthcare system, leading to unclear guidelines regarding how to facilitate a proper transition from pregnancy to the postpartum period. HCPs communicated risk differently to GDM-affected women, and there was no consensus on how implications of GDM should travel across sectors, which led to inconsistencies in the management of women with GDM, i.e., from the obstetric departments to the GP and health visitors. Taken together, our results suggest that HCPs find it challenging and report barriers in providing a coherent care pathway for women with current and prior GDM.

### 4.1. Communicating Risk after Pregnancy

GDM is a complex condition, which demands interprofessional collaboration to provide a coherent care pathway to ensure that preventive care and thus long-term health promotion is prioritized. The need for extended postpartum care for women with prior GDM has been underlined by many studies [24] and emphasized by the women themselves [17,25]. Thus, there seems to be a lost opportunity to support women in healthy lifestyles after a GDM-affected pregnancy to facilitate postpartum weight loss, improve their cardiometabolic profile and ultimately lower their risk for recurrent GDM and early type 2 diabetes onset. 

In a qualitative study from Sweden, midwives focused on the delivery and transition to parenthood when managing women with GDM, distancing them from complications related to pregnancy [26]. In our study, obstetricians and diabetes nurses tried to uphold the motivation for women with GDM to stay healthy during pregnancy; however, this motivation did not seem to be sustained after delivery due to other priorities in GP consultations and a lack of awareness by health visitors. Other qualitative studies suggest that a reduced risk perception among women with prior GDM is a barrier to engaging in healthy activities postpartum [27,28]. Thus, GPs and health visitors attending to women with prior GDM should intensify their communication strategies aimed at increasing risk perception to motivate health promoting behaviors. Nevertheless, in a study of Danish GPs, it was found that addressing health-related issues, such as smoking and obesity, can lead to mistrust of the GP’s authority, creating less incentive and motivation for the patient to uptake health recommendations [29]. Thus, even though GPs may value and want to integrate preventive care in their practices, they face challenges in providing preventive care. Furthermore, as health visitors are responsible for family health promotion postpartum in Denmark, GPs may not consider themselves responsible for such preventive practices [12].

### 4.2. Creating Awareness around GDM 

In addition to challenges in risk communication, we found that unclear guidelines and geographical distance make it difficult for GPs and HCPs in obstetric departments to ensure a coherent transfer of information across health sectors. In a study from the UK, diabetologists, obstetricians and GPs, representing primary and secondary care for women with GDM, reported that the National Institute for Health and Clinical Excellence (NICE) guidelines on follow-up of women with prior GDM [30] was not followed systematically [31] Thus, increasing awareness around guidelines for diabetes prevention among HCPs may not solve the problem singlehandedly. O’Reilly suggests improving cross-sectoral knowledge transfer while providing social support for women with prior GDM to engage them in healthy behaviors as a solution to strengthen type 2 diabetes prevention [32] In Denmark, health visitors have one of the most central health-promoting roles as they attempt to meet complex healthcare needs in their outreach to families. For this reason, health visitors or other HCPs with similar backgrounds and/or resources, e.g., public health nurses, may be better suited to optimize health promotion practice for women with prior GDM. A systematic review of collaboration between health visitors and midwives found that a big advantage of collaboration was the positive effect on attending to families with more complex needs [33] Thus, health visitors may hold an essential role in supporting women with prior GDM with their broad focus on health and their detachment from the clinic-based healthcare system. Another solution could be to restructure the healthcare system which most likely would reframe professions, creating new incentive structures and reward systems for collaboration [34] Lastly, an alternative to reorganization is to raise awareness about boundaries between HCPs to change the focus from a merely treating healthcare system to a health-promoting one [35].For example, having midwives deliver the information about a GDM-affected pregnancy to health visitors may enable them to address the need for health behaviors, promote postpartum weight loss and reduce the risk of type 2 diabetes.

### 4.3. Strengths and Weaknesses of the Study

Due to the significance of collaboration across sectors in GDM care, it is a major strength of this study that we managed to include HCPs from all sectors providing care to women with GDM during and after pregnancy. This approach was key in the recruitment of participants to ensure a broad variety of perspectives on the GDM care pathway. It allowed us to identify unclear guidelines across sectors along with limited resource allocation to GDM care by several providers relevant for planning of long-term follow-up of women with prior GDM. Additionally, it was a strength to use STC, as it enabled the identification of core themes and essential characteristics of the interview data. Third, focus group discussions structured the interview guides by exploiting group dynamics [36], and testing the hypothesis of whether risk communication was challenging to health visitors. Accordingly, the interview guide was adapted to the interviews with HCPs, allowing new themes to arise and for HCPs to provide suggestions for changes in care [37]. We applied a qualitative approach to capture the provider perspectives of GDM care. No other study has involved providers that engage with women with GDM pre- and post- delivery to investigate the care pathway for GDM during and after pregnancy in the Danish healthcare system. The implications of this study pinpoint the direction for future practice changes and suggest how HCPs could be involved in advancing care in the postpartum period through better communication practices and/or restructuring the care pathway for women with GDM entirely.

The recruitment of informants was based on purposive sampling and the snowball method, which may have introduced less variation in subjects. The HCPs who agreed to take part may have been prone to emphasize the need for extended care in the postpartum period for women with prior GDM compared to other HCPs. By including HCPs from different professions, we were able to obtain diverse insights on topics that the participants had in common [18].

The HCPs were recruited from obstetric departments in large hospitals in Copenhagen and Aarhus, making the findings more generalizable to hospitals where multiple HCPs are involved in GDM care. Inconsistencies in GDM care have been identified across countries. However, generalizing the results outside Denmark is questionable, especially considering that Denmark has a universal tax-funded public healthcare system [38]. Though we included HCPs from all sectors, we were unable to recruit endocrinologists, and dieticians, who also work closely with women with GDM. Including their perspectives would have strengthened the rigor by representing perspectives from all HCPs engaged in GDM care. 

### 4.4. A Need to Reorganize Care for Women with Prior GDM

With this study, we identified barriers and possibilities for improvement of cross-sectoral collaboration for women with GDM during and after pregnancy. Our study shows that the responsibility to ensure a coherent preventive treatment pathway across sectors for women with GDM does not lie with one specific HCP in the Danish healthcare system. Therefore, systematic, coherent cross-sectoral communication and transparency is crucial, especially since screening and prevention of type 2 diabetes after GDM seems to be cost-effective or cost-saving [39,40] Unfortunately, the findings from our study highlight that collaboration between various providers across sectors is incoherent, which leads women to be inadequately supported after a GDM-affected pregnancy. A potential strategy to redeem this may be by introducing case managers or assigning midwives or health visitors to coordinate and integrate an increased prevention focus in the care pathway after delivery. This approach could help to ensure coherence in hospital settings and across sectors for women with prior GDM and potentially strengthen action toward long-term diabetes prevention.

## 5. Conclusions

In this study, we investigated HCPs’ perspectives on the cross-sectoral GDM care pathway during and after pregnancy. Three themes were identified: (1) “professional identities” framed various communication practices, (2) “unclear guidelines on type 2 diabetes prevention after GDM” contributed to uncertainty about tasks and responsibilities, and (3) “cross-sectoral collaboration” was lacking due to inconsistent knowledge transfers across sectors. The main implication of our results is that a strong and improved cross-sectoral collaboration is needed for GDM care, as it is managed by multiple HCPs with different identities and agendas during and after pregnancy. We suggest rethinking the treatment pathway of women with GDM by ensuring better reporting and collaboration between HCPs after pregnancy. Further, we recommend incorporating a long-term prevention perspective in the period after pregnancy including support to postpartum weight loss and healthy behaviors. More attention should be directed toward structures that ease cross-sectoral communication, transparent guidelines and tailored communication strategies for HCPs handling women with current and prior GDM.

## Figures and Tables

**Table 1:** Trajectory of Danish gestational diabetes mellitus (GDM) care during and after pregnancy compared to standard care for non-GDM-affected pregnant women.

	Non-GDM-Affected Pregnancy		GDM-Affected Pregnancy	
	Pregnancy	After Delivery	Pregnancy	After Delivery
General practitioner	3 consultations	2 consultations	3 consultations	3 consultations
Diabetes nurse	-	-	3–5 consultations	0–1 ** consultations
Endocrinologist *	-	-	0–5 consultations *	
Obstetrician		-	3–5 consultations	-
Dietician	-	-	3 consultations	0–1 consultation
Midwife	3–5 consultations	-	4–6 consultations	-
Health visitor	-	5 home visits	0–1 consultation ***	5 home visits
Total visits	6–8 consultations	7 consultations	16–28 consultations	8–10 consultations
Extra GDM consultations			10–20 consultations	1–3 consultations

* The endocrinologist is involved if the woman requires treatment with insulin (20–25% of cases in Denmark). ** The responsibility to conduct the follow-up oral glucose tolerance test instead is shared between the general practitioner and the diabetes nurse. *** The woman with GDM can be offered a home visit by the health visitor if the woman is diagnosed with GDM in pregnancy.

**Table 2:** Background characteristics of participating healthcare professionals.

Method	Interview Person	Occupation	Seniority	Sector
Focus group 1	A	Health visitor	>15 years	Municipality
	B		10–14 years	
	C		<5 years	
	D		<5 years	
Focus group 2	E		>15 years	
	F		5–9 years	
	G		<5 years	
	H		<5 years	
Semi-structured interview	I	Midwife	>15 years	Obstetric department, regional
	J		10–14 years	
	K		>15 years	
	L		5–9 years	
	N	Obstetrician	5–9 years	
	O		10–14 years	
	P	Diabetes nurse	>15 years	
	Q	General practitioner	>15 years	General practice
	R		>15 years	

**Table 3:** Major themes and key points identified in focus groups and interviews.

Major Themes	Key Points
Professional identities	GPs, midwives and health visitors experienced difficulties in addressing health behaviors after GDM in consultations with women diagnosed with GDM, whereas obstetricians and a diabetes nurse saw it as part of their job to talk about risks related to GDM in consultations. Midwives did not want to blame the women by addressing the GDM-related health risks for the child; health visitors were limited in their knowledge of the need for health behavior change after GDM, and GPs focused on other topics. Addressing health behaviors depended on both women’s and HCP’s individual preferences.
Unclear guidelines on type 2 diabetes prevention after GDM	When women have given birth and no longer have GDM, the follow-up care after pregnancy became ill-prioritized. HCPs perceived the lack of care provided to women with prior GDM as unsupportive. The diagnosis of GDM is disregarded by HCPs after pregnancy, underlining the need for clear guidelines on how to address the implications of GDM after delivery.
Cross-sectoral collaboration	According to HCPs, the different communication strategies among HCPs caused women to perceive their GDM diagnosis as either very demanding or insignificant. GPs reported not being provided with sufficient information on the women’s treatment course from the obstetric departments, and health visitors did not receive information on whether the woman had been diagnosed with GDM. Limited information flow between HCPs were perceived to contribute to poor cross-sectoral collaboration and follow-up after birth.

## Data Availability

The data that support the findings of this study are available from the corresponding author, but restrictions apply to the availability of these data which were used under license for the current study and so are not publicly available. Data are however available from the authors upon reasonable request and with permission of the participants in the study.

## Supplementary Materials

The following are available online at https://www.mdpi.com/2077-0383/10/4/843/s1; Table S1: Semi-structured, single interview guide.
