# Supplementary material for: Healthcare Professionals’ Perspectives on the Cross-Sectoral Treatment Pathway for Women with Gestational Diabetes during and after Pregnancy—A Qualitative Study
Source: J Clin Med. 2021 Feb 18;10(4):843. doi: 10.3390/jcm10040843 (PMC7923110; doi:10.3390/jcm10040843)
Supplement: Supplementary file 1 [file jcm-10-00843-s001.pdf]

Table S1: Semi-structured, interview guide

Research Questions	Interview Questions
Organisational structures<br><br>What relevant contextual factors affect your work with women with gestational diabetes?	Can you tell me a little about your work and work-related assignments? For how long have you worked here? For how long have you been engaging with women with gestational diabetes?<br><br>Do you think that gestational diabetes is prioritised in your profession?<br><br>Can you tell me how your interaction with women with pregnancy diabetes proceeds the first time you see them? How many times do you see them on average?<br><br>Are you talking to women after they have had a diabetic pregnancy? And what do you talk about?<br><br>What do you prioritise in the conversation with women with gestational diabetes? Why?<br><br>Do you experience that you can control and prioritise freely in the consultation in relation to what you think is important?<br><br>Can you describe whether you believe that others with the same background have the same flexibility or if you experience that you prioritise differently than other healthcare professionals within the same profession?
Experienced barriers for women with gestational diabetes under and after birth<br><br>Which barriers do the healthcare professionals experience in the treatment of women with gestational diabetes and are there any differences across professions?	What is your experience of what women with gestational diabetes are doing lifestyle wise after birth?<br><br>Do you think the women leave the clinic with sufficient knowledge about the implications of having had diabetes in pregnancy?<br><br>Do you feel that women face problems living a healthy lifestyle after birth? What do you think are the main barriers for women to maintain a healthy lifestyle both under and after pregnancy?<br><br>Do you think that women experience stigmatisation or labelling in connection with gestational diabetes?<br><br>Do you think it is hard to talk to women about e.g. overweight or that they should eat healthier and exercise more?<br><br>What do you think are the most facilitating factors for women to engage in health-related behaviours under and after pregnancy?
Cross-sectoral collaboration<br><br>How do the healthcare professionals experience cross-sectoral collaboration in connection to the treatment course of women with gestational diabetes?	Can you describe how women with gestational diabetes are followed up after pregnancy?<br><br>What is your responsibility in the course of the woman during and after pregnancy?<br><br>What is your impression of how much women interact with other healthcare professionals after they have given birth?

	Which other healthcare professions do you contact when planning the treatment course and follow-up after birth? Why these? And who do you work best with?<br><br>What subjects do you feel most responsible for in guiding the woman or following her after birth? And why?<br><br>What is your own motivation for working across sectors or with other professions?<br><br>What restrictions or facilitating factors do you think determine whether there exists a good or bad collaboration?
Intervention possibilities<br><br>How do healthcare professionals experience opportunities to integrate an intervention focusing on healthy lifestyle for women after gestational diabetes?	What do you think about the possibility of introducing an intervention that focuses on preventing T2DM after gestational diabetes through lifestyle changes like healthy diet and exercise?<br><br>What is needed to initiate such an intervention?<br><br>Do you find it relevant for you and your colleagues to talk about healthy lifestyle with the women after GDM? Why / Why not?<br><br>Do you feel that your specialty is lacking in competencies to handle tasks in a possible intervention?<br><br>Which healthcare professions should be involved? Why? Who should be the primus engine?<br><br>Do you think these professions would be motivated to engage in a lifestyle intervention? Why?<br><br>Do you think there is a lack of resources to start initiating such an intervention? If yes, which?

"Is there anything you would like to add to this topic about women with gestational diabetes, which we have not talked about?"

"So, at last, is there anything that you do not want to be cited for?"

Thank you for your participation!
